# NeuroD2 controls inhibitory circuit formation in the molecular layer of the cerebellum

**DOI:** 10.1038/s41598-018-37850-7

**Published:** 2019-02-05

**Authors:** Alexander Pieper, Stephanie Rudolph, Georg L. Wieser, Tilmann Götze, Hendrik Mießner, Tomoko Yonemasu, Kuo Yan, Iva Tzvetanova, Bettina Duverge Castillo, Ulli Bode, Ingo Bormuth, Jacques I. Wadiche, Markus H. Schwab, Sandra Goebbels

**Affiliations:** 1 Max-Planck-Institute of Experimental Medicine, D-37075 Goettingen, Germany; 2 Department of Neurobiology and Evelyn McKnight Brain Institute, University of Alabama at, Birmingham, AL 35294 USA; 3 Present Address: Department of Neurobiology, Harvard Medical School, Boston, MA 02115 USA; 4 Present Address: Charité-Universitätsmedizin Berlin, Institute of Cell Biology and Neurobiology, NeuroCure Cluster of Excellence, D-10115 Berlin, Germany; 5 Present Address: Cellular Neurophysiology, Hannover Medical School, D-30625 Hannover, Germany; 6 Center for Systems Neuroscience (ZSN), Hannover, Germany

## Abstract

The cerebellar cortex is involved in the control of diverse motor and non-motor functions. Its principal circuit elements are the Purkinje cells that integrate incoming excitatory and local inhibitory inputs and provide the sole output of the cerebellar cortex. However, the transcriptional control of circuit assembly in the cerebellar cortex is not well understood. Here, we show that NeuroD2, a neuronal basic helix-loop-helix (bHLH) transcription factor, promotes the postnatal survival of both granule cells and molecular layer interneurons (basket and stellate cells). However, while NeuroD2 is not essential for the integration of surviving granule cells into the excitatory circuit, it is required for the terminal differentiation of basket cells. Axons of surviving NeuroD2-deficient basket cells follow irregular trajectories and their inhibitory terminals are virtually absent from Purkinje cells in *Neurod2* mutants. As a result inhibitory, but not excitatory, input to Purkinje cells is strongly reduced in the absence of NeuroD2. Together, we conclude that NeuroD2 is necessary to instruct a terminal differentiation program in basket cells that regulates targeted axon growth and inhibitory synapse formation. An imbalance of excitation and inhibition in the cerebellar cortex affecting Purkinje cell output may underlay impaired adaptive motor learning observed in *Neurod2* mutants.

## Introduction

The cerebellum controls motor behavior and adaptive motor learning. Purkinje cells serve as the sole output neurons of the cerebellar cortex and provide inhibitory input to neurons of the deep cerebellar and vestibular nuclei^1,2^. Purkinje cells have prominent intrinsic pacemaker activity and fire complex and simple spikes by integrating excitatory input from climbing fibers (CF) and parallel fibers (PF), respectively, and inhibitory input from molecular layer interneurons (MLIs)^3^. During cerebellar development CF arise from glutamatergic neurons in the inferior olivary nucleus of the ventral brainstem. After a period of synapse elimination in early postnatal development, a single CF innervates the proximal dendrite of each Purkinje cell in the mature animal^4^. In contrast, PF are derived from glutamatergic granule cells located within the cerebellar cortex. Granule cells originate from precursor cells in the rhombic lip marked by the expression of the proneural bHLH transcription factor Atoh1^5,6^. Following tangential migration and a proliferative stage in the external granule cell layer (EGL), postmitotic immature granule cells initiate outgrowth of axons and migrate radially through the molecular layer (ML) to their final positions in the internal granule cell layer (IGL) during early postnatal stages^7^.

A distinct precursor pool in the primitive cerebellar neuroepithelium, which expresses the proneural bHLH protein Ptf1a^8^, gives rise to all GABAergic neurons of the cerebellum. This includes Purkinje cells, the major neuronal subtype derived from the Ptf1a^+^ ventricular zone, in addition to Golgi cells and MLI lineages. MLI precursors continue to proliferate postnatally in the prospective white matter, migrate towards the nascent ML, and subsequently undergo extensive differentiation, including targeted axon growth and synapse formation^9–11^. Based on morphological criteria, two types of GABAergic MLIs have been described. Stellate cells in the outer ML preferentially innervate Purkinje cell dendrites while basket cells in the deeper ML contact the perisomatic compartment of Purkinje cells^12,13^. However, it is not finally settled whether these interneurons represent two distinct cell types or one functionally continuous population^14^. MLIs control Purkinje cell output by providing feed-forward inhibition in response to PF and CF activation^15–19^. MLIs are therefore essential to cerebellar processing, but the transcriptional mechanisms that regulate the diversification of MLIs and their differentiation remain incompletely understood^11^.

Transcription factors from the bHLH family frequently act in cascades during development. The proneural bHLH factors Atoh1 and Ptf1a are necessary and sufficient to specify granule cell and MLI lineages, respectively^8,20,21^, consistent with a function of additional bHLH proteins in these cell lineages during later developmental stages. Indeed, NeuroD1 and NeuroD2, members of the NeuroD subfamily of neuronal bHLH proteins, have been implicated in the timing of ‘transit amplification’ of granule cells in the EGL^22^ and the support of granule cell survival in the IGL^23,24^. NeuroD subfamily proteins were also shown to regulate neurite stratification of inhibitory amacrine cells in the retina^25^ and peptidergic differentiation of inhibitory neurons in the dorsal spinal cord, downstream of Ptf1^26^. Since expression of the *Neurod2* gene was observed in MLIs^24,27^, members of the NeuroD subfamily represent plausible candidate transcription factors to regulate excitatory and inhibitory circuit formation in the cerebellum.

Here, we show that deletion of *Neurod2* in mice affects granule cell survival during a critical postnatal period, but is not essential for the assembly of granule cell circuitry. In contrast, NeuroD2 deficiency not only decreases MLI survival, but also impedes basket cell axogenesis and synapse formation onto Purkinje cells. We therefore hypothesize that an imbalance of excitatory and inhibitory neurotransmission in the cerebellar cortex could contribute to impaired motor learning in *Neurod2* mutants.

## Results

### Generation of viable *Neurod2* null mutant mice

**Figure 1:**
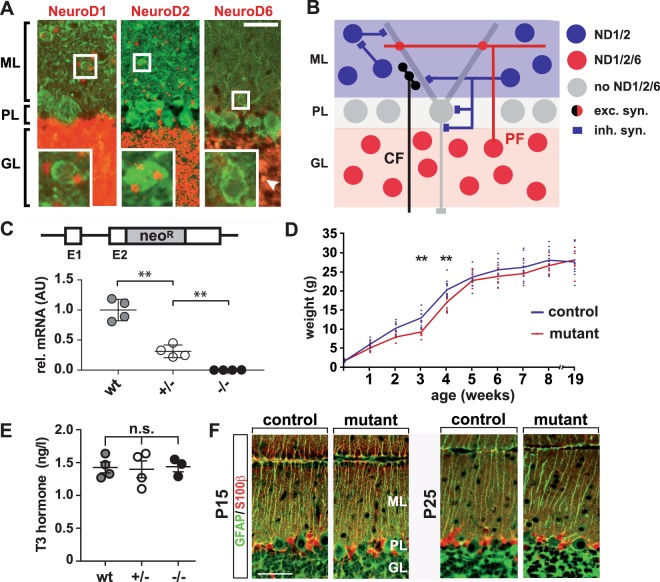
Long-term survival of *Neurod2*^−/−^ *mutants on a 129/SvJ genetic* background. (**A**) Expression of NeuroD family members in the cerebellar cortex. (left panel) *Neurod1* is expressed in MLIs (see magnification of boxed area) and granule cells, but not in Purkinje cells of *Neurod1-lacZ* ‘knock in’ mice at P18. (middle panel) Similarly, *Neurod2* is expressed in MLIs and granule cells, but not in Purkinje cells of *Neurod2-lacZ* ‘knock in’ mice at P20. *Neurod1* and *Neurod2* expression was identified by X-gal histochemistry (displayed in red pseudocolor) followed by fluorescent immunostaining for PV (green) to identify MLI and Purkinje cells. (right panel) Scattered *Neurod6* expression in postmigratory granule cells (arrowhead), but no expression in MLI and Purkinje cells at P18. *Neurod6* expression was identified by Cre-mediated activation of a td-tomato reporter in NEXCre*td-tomato double transgenic mice. Scale bar, 25 µm. (**B**) Schematic of excitatory and inhibitory connections and the NeuroD family ‘expression code’ in the cerebellar cortex. CF, Climbing fibers; exc., excitatory; GL, granule cell layer; inh., inhibitory; ML, molecular layer; ND, NeuroD; PL, Purkinje cell layer; PF, Parallel fibers. (**C**) Quantitative RT-PCR analysis demonstrates absence of *Neurod2* mRNA from the cerebellum of *Neurod2*^−/−^ mutants (−/−) at 3 months of age. Results show mean fold changes normalized to wildtype controls (set to 1; n = 4 per genotype). (**D**) After backcrossing to a 129/SvJ genetic background *Neurod2*^−/−^ mutants (n = 9 males) display a transient delay in weight gain (week 1–4) compared to heterozygous controls (n = 11 males), but catch up with age (mean ± SD). (**E**) Serum T3 levels are not altered in Neurod2^+/−^ and Neurod2^−/−^ mutants compared to wildtype at P7 (n = 3–4 per genotype; n.s., not significant). (**F**) Bergmann glia cells in *Neurod2*^−/−^ mutants show a normal morphology with respect to the localization of S100β^+^ cell bodies and the number and structure of GFAP^+^ radial processes. Immunostaining on sagittal cerebellar sections at P15 (left) and P25 (right). Scale bar, 25 µm.

*Neurod2* expression was previously reported in the ML of the cerebellar cortex, most likely derived from MLIs^24^. Several co-expressed NeuroD family members often serve partly overlapping functions in CNS neurons^28^. Therefore, we determined expression patterns of NeuroD1, NeuroD2, and NeuroD6 using sensitive reporter mouse lines. X-gal histochemistry combined with immunostaining for parvalbumin (PV) in *Neurod2-lacZ* ‘knock in’ reporter mice^24^ confirmed *Neurod2* expression in MLIs at P20. In addition, Neurod2 was prominently expressed in granule cells, but absent from Purkinje cells (Fig. 1A). A corresponding analysis of *Neurod1*-lacZ ‘knock in’ reporter mice^29^ revealed similar expression of *Neurod1* in granule cells and MLIs, which was absent from Purkinje cells (Fig. 1A). In *Nex*-Cre ‘knock in’ mice^30^ harboring a red fluorescent Cre reporter^31^, scattered *Neurod*6 expression was present in granule cells, whereas *Neurod6* expression was absent from Purkinje cells and MLIs (Fig. 1A). These findings identify distinct expression profiles of NeuroD family members in granule cells and MLIs, providing excitatory and inhibitory input, respectively, to NeuroD family-negative Purkinje cells (Fig. 1B).

These expression profiles suggest partially overlapping NeuroD1 and NeuroD2 functions in granule cells and MLI, which could be investigated by a loss-of-function approach in mice. We recently generated *Neurod2* mouse mutants^28^ (Fig. 1C), which showed pronounced growth retardation after birth on a mixed C57Bl/6 × 129/Sv genetic background (not shown). Most *Neurod2* null mutants (*Neurod2*^−/−^) died before the age of 4 weeks during breeding to a C57Bl/6 genetic background, similar to *Neurod2-lacZ* mice^24^. Therefore, we backcrossed *Neurod2*^−/−^ mutants into a 129/SvJ genetic background, a strategy previously used to circumvent neonatal lethality of *Neurod1* null mutants^32^. Premature death of *Neurod2*^−/−^ mutants was no longer observed after backcrossing into a 129/SvJ genetic background, i.e. breedings of heterozygous (*Neurod2*^+/−^) with homozygous *Neurod2*^−/−^ mutants produced 47% (53 out of 113 pups) *Neurod2*^−/−^ mutants at two months of age. Quantitative real time-PCR (Fig. 1C) and Northern blotting (not shown) confirmed absence of *Neurod2* transcripts from the cerebellum of adult *Neurod2*^−/−^ mutants. A transient delay in weight gain persisted even after 4 backcrosses, but *Neurod2*^−/−^ mutants caught up after 4 weeks of age (Fig. 1D). Growth retardation of *Neurod2-lacZ* mutants in a mixed C57Bl/6 × 129/Sv background was associated with reduced serum levels of thyroid hormones^33^. However, analysis of thyroid hormones in *Neurod2*^−/−^ mutants in a 129/SvJ background revealed unaltered serum levels of T3 at P7 (Fig. 1E) and T3/T4 at P20 (data not shown). Disturbed organization of radial Bergmann glia is a hallmark of thyroid hormone deficiency^34^. Consistent with a normal thyroid hormone status, we observed no changes in radial Bergmann glia formation in the cerebellum of *Neurod2*^−/−^ mutants (Fig. 1F). Thus, backcrossing to a 129/SvJ genetic background restored a normal thyroid hormone status and provided a strategy to investigate the outcome of *Neurod2* deficiency during postnatal and adult stages.

### NeuroD2-deficient granule cells terminally differentiate and functionally integrate into the cerebellar circuit

**Figure 2:**
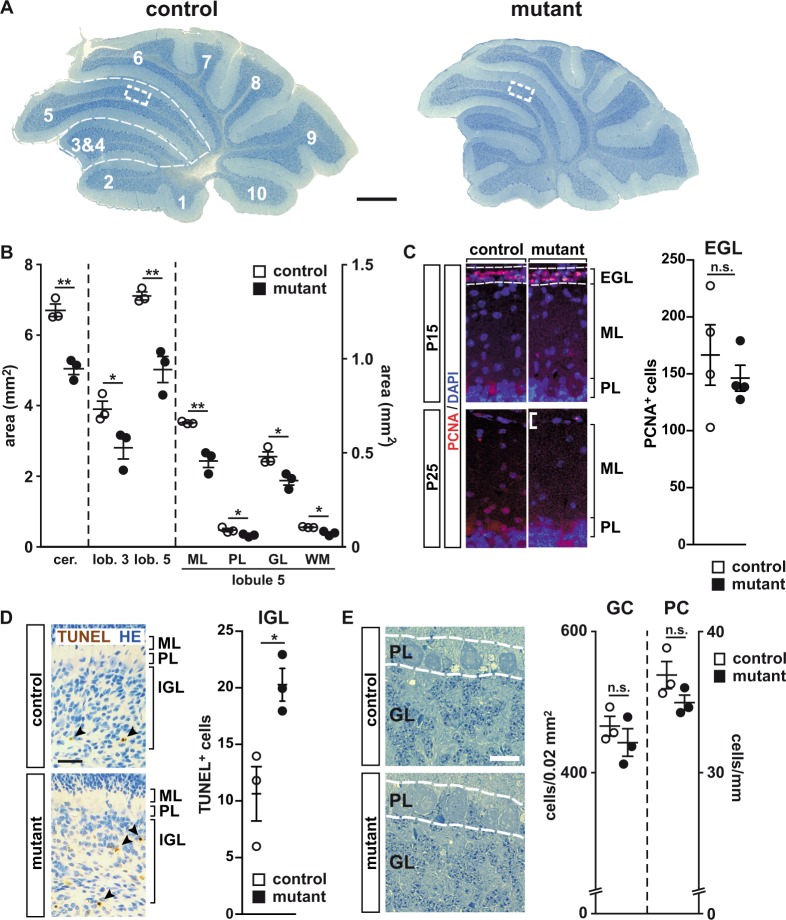
A role of NeuroD2 in perinatal survival of postmitotic and postmigratory granule cells. (**A**) Decreased cerebellar size in *Neurod2*^−*/*−^ mutants compared to heterozygous controls at P30. Toluidine-blue staining of sagittal sections. Scale bar, 500 µm. (**B**) Area reduction equally affected lobules 3 and 5, as well as individual layers in lobule 5. (n = 3 per genotype; results, mean ± sem.; two-tailed T-Test with Welch correction). cer., total cerebellum; GL, granule cell layer; lob. 5, total lobule 5; ML, molecular layer; PL, Purkinje cell layer; WM, white matter. (**C**) Proliferation and migration of granule cell progenitors is not delayed in *Neurod2*^−*/*−^ mutants. Fluorescent immunostaining on sagittal cerebellar sections for PCNA^+^ progenitors in the EGL at P15 (dashed lines), which are virtually absent in both genotypes at P25. Scale bar, 50 µm. n = 4 per genotype. (**D**) Increased number of apoptotic granule cells in the IGL of *Neurod2*^−/−^ mutants at P5 (arrowheads) compared to heterozygous controls. TUNEL assay on sagittal sections. Scale bar, 50 μm. n = 3 per genotype. (**E**) Purkinje cell and granule cell densities are not altered in lobule 5 of *Neurod2*^−*/*−^ mutants compared to controls. Images show detail of PL and GL located within a 0.02 mm^2^ region of interest in the dorsal region of lobule 5 (dashed boxes in **A**). Note that Purkinje cell ‚density’ is displayed as cells per PL length (in mm), in contrast to granule cell density displayed as cells per GL area (mm^2^) (n = 3 per genotype; results, mean ± sem.; two-tailed T-Test with Welch correction). Scale bar, 10 µm.

**Figure 3:**
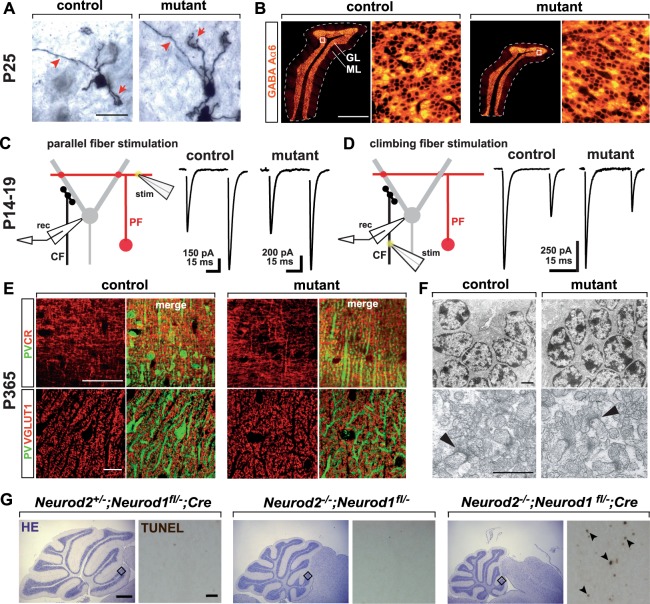
Surviving NeuroD2-deficient granule cells terminally differentiate and functionally integrate into excitatory cerebellar circuits. (**A**) Granule cells in *Neurod2*^−/−^ mutants extend claw-like telodendria (arrow) and axons projecting towards the ML (arrowhead). Golgi impregnation of sagittal sections (P25). Scale bar, 20 µm. (**B**) Fluorescent immunostaining for GABA Aα6 receptor on sagittal sections (lobule 5) from *Neurod2*^−/−^ mutants and controls (P25). (Right panels) magnification of boxed area in left panels. Scale bar, 600 µm. (**C**) Left: Schematic of PF EPSC recordings from Purkinje cells. PFs were stimulated in the ML (stim) while Purkinje cells were held in whole-cell voltage clamp (rec). Right: Representative traces of PF EPSCs from controls and *Neurod2*^−/−^ mutants. Paired-pulses reveal similar EPSC2 facilitation compared to EPSC1. (**D**) Left: Schematic of recordings of CF EPSCs from Purkinje cells. CFs were stimulated in the GL (stim) while Purkinje cells were held in whole-cell voltage clamp (rec). Right: Representative traces of CF EPSCs from control and *Neurod2*^−/−^ mutants. Paired-pulses reveal similar depression of EPSC2 compared to EPSC1. (**E**) Normal number and distribution of PFs and their terminals in *Neurod2*^−/−^ mutants compared to controls. Fluorescent immunostaining for Purkinje cell dendrites (PV) and PFs (calretinin, CR; or VGLUT1) (age 1 year). Note differences in PV staining due to loss of PV^+^ MLIs in *Neurod2*^−/−^ mutants. Scale bars, 20 µm. (F) (Upper panels) Electron microscopy of granule cells in *Neurod2*^−/−^ mutants exhibits a similar distribution of nuclear heterochromatin compared to controls (age 1 year). Scale bar, 1.7 µm. (Lower panels) Ultrastructure of PF/Purkinje cell synapses (arrowheads) in *Neurod2*^−/−^ mutants compared to controls (age 1 year). Scale bar, 1 µm. (**G**) Overlapping NeuroD1 and NeuroD2 functions in postmigratory granule cell survival. (left) Normal cerebellar size and absence of granule cell apoptosis (TUNEL assay of GL in lobule 2) at P21 in conditional *Neurod1* null mutants (NeuroD1 deficiency in postmigratory granule cells) with one wildtype copy of *Neurod2* (*Neurod2*^+/−^; *Neurod1*^fl/−^; Cre). (Middle) *Neurod2*^−/−^ mutants with one functional copy of *Neurod1* (*Neurod2*^−/−^; *Neurod1*^fl/−^) display reduced cerebellar size similar to *Neurod2*^−/−^ single mutants (see **B**). Note that granule cell apoptosis has ceased by P21. (right) Reduced cerebellar size and granule cell apoptosis (arrowheads) in *Neurod1/2 double mutants* (*Neurod2*^−/−^; *Neurod1*^fl/−^; Cre). Scale bars, 500 µm (H&E stainings); 30 µm (TUNEL).

Total cerebellar size was reduced by ~30% in *Neurod2*^−*/*−^ mutants at P30 compared to heterozygous *Neurod2*^+/−^ mice (in the following referred to as controls) when measured on parasagittal sections of the cerebellar vermis (Fig. 2A,B). A quantification of total area sizes of lobules 3 and 5, as well as individual layers within lobule 5 (molecular layer, ML; Purkinje cell layer, PL; granule cell layer, GL; white matter, WM), suggested that size reduction in *Neurod2*^−*/*−^ mutants affected all lobules tested and individual layers within those lobules to a similar extent (Fig. 2B). As a result, the percentage of lobule 5 area covered by individual layers was unaltered in *Neurod2*^−*/*−^ mutants. Fluorescent immunostaining at P15 demonstrated similar numbers of PCNA-positive cells in the EGL of *Neurod2*^−/−^ mutants and controls (control, 166 ± 26.43; mutant, 145.6 ± 11.29; n = 4 each; p = 0.5049; Fig. 2C), suggesting no changes in the proliferation of granule cell progenitors in the EGL or a delay in radial granule cell migration from the EGL to the IGL in *Neurod2*^−*/*−^ mutants. However, in agreement with previous findings^24^, TUNEL staining revealed an increased granule cell apoptosis in the IGL of *Neurod2*^−/−^ mutants at P5 (control, 10.67 ± 2.404; mutant, 20.33 ± 1.453; n = 3 each; p = 0.0263; Fig. 2D), suggesting that size reduction of the GL in adult mutants results from the loss of postmitotic and postmigratory granule cells in the IGL. When quantified in a dorsal region of lobule 5 (marked by dashed boxes in Fig. 2A), granule cell density was not significantly reduced in *Neurod2*^−/−^ mutants at P30 (Fig. 2E). Even when measured in dorsal lobule 5 at 1 year of age, granule cell density in *Neurod2*^−/−^ mutants was not significantly changed (control, 417 ± 36/0.02 mm^2^; mutant, 401 ± 18/0.02 mm^2^; n = 3 each; p = 0.99), and we found no evidence for granule cell apoptosis or secondary granule cell degeneration in *Neurod2*^−/−^ mutants after P20 (Fig. 3F,G). Interestingly, the total number of NeuroD2-negative Purkinje cells was also reduced in lobule 5 of Neurod2^−/−^ mutants at P30 (control, 192 ± 6; mutant 149 ± 6; n = 3 each; p < 0.05). However, combined with reduced PL ‘length’ (measured in mm), the number of Purkinje cells per mm PL was not significantly reduced (Fig. 2E). These data demonstrate that NeuroD2 is required for the survival of granule cells during a critical postnatal period, but suggest that NeuroD2 is not essential for the long-term maintenance of granule cells.

Next, we tested the impact of NeuroD2-deficiency on the functional integration of glutamatergic cerebellar inputs. Golgi staining at P25 identified granule cell dendrites with claw-like telodendria, a feature of differentiated granule cells, in *Neurod2*^−/−^ mutants. Similarly, we observed no changes in projections of granule cell axons towards the ML in *Neurod2*^−/−^ mutants (Fig. 3A), suggesting intact PF trajectories and Purkinje cell innervation. Further support for normal postsynaptic development came from immunostaining for the α6 subunit of the GABA A receptor, a marker of terminally differentiated granule cells, which revealed normal expression at postsynaptic sites (Fig. 3B), in line with intact GABAergic input by Golgi cells onto granule cells in cerebellar glomeruli. To investigate the functional integration of NeuroD2-deficient granule cells into the cerebellar circuit, we recorded PF-mediated EPSCs from Purkinje cells in acute cerebellar slices using whole-cell patch clamp (Fig. 3C). Consistent with anatomical data, PF-Purkinje cell synapses were functional in *Neurod2*^−/−^ mutants, and PF-EPSCs facilitated to a similar extent as in controls (EPSC2/EPSC1; control, 1.51 ± 0.13; mutant, 1.58 ± 0.03; n = 5 each; p = 0.61; Fig. 3C), showing functional integration of NeuroD2-deficient granule cells into the cerebellar circuit. We also evaluated PF-EPSC kinetics to assess potential changes in transmitter release (PF: control rise, 0.50 +/− 0.06 ms, n = 5, mutant rise 0.57 +/− 0.6 ms, n = 6, p = 0.38; control decay: 3.2 +/− 0.7 ms, mutant decay: 3.0 +/− 0.3 ms, p = 0.73). The similar kinetics of control and mutant PF-EPCSs suggest that deletion of NeuroD2 does not significantly alter the time course of transmitter release at the PF-PC synapse. The similar EPSC kinetics are also consistent with the absence of gross changes in Purkinje cell morphology of NeuroD2 mutants. To test for differences in PF density we also stimulated PF inputs with increasing stimulus intensities. The slopes of the input-output curves of PF-EPSCs derived from 4 stimulus intensities (0, 3, 6 and 9 mV) for control and mutant were 0.11 and 0.12, respectively, and were not significantly different from each other (p = 0.91, n = 4 each), suggesting the PF density is similar in mutant and control. Purkinje cells also receive powerful excitatory input from CFs originating in the inferior olive. Postnatal elimination (~P14) of transiently established multiple CF inputs marks Purkinje cell maturation and requires normal PF development^35,36^. To test for multiple CF inputs, CF-EPSCs in Purkinje cells were evoked with increasing stimulus intensities. Step-wise increase of stimulation intensity revealed normal maturation of CF inputs onto Purkinje cells in *Neurod2*^−/−^ mutants (data not shown). Additionally, CF-EPSC time courses were similar in controls and *Neurod2*^−/−^ mutants (rise time; control, 0.47 ± 0.07 ms; mutant, 0.46 ± 0.05 ms, p = 0.86; decay time; control, 4.0 ± 0.4 ms; mutant, 3.5 ± 0.2 ms, p = 0.31, n = 8 and n = 7, respectively), ruling out the presence of immature somatic CF synapses with much faster kinetics^37^ in *Neurod2*^−/−^ mutants. Finally, CF-EPSC paired-pulse ratios were also unchanged in *Neurod2*^−/−^ mutants (Fig. 3D, control, 0.49 ± 0.04, n = 8; mutant, 0.44 ± 0.03, interstimulus interval = 50 ms; n = 7; p = 0.2) indicating similar vesicle release probabilities. Together, these results suggest that excitatory synaptic input onto Purkinje cells provided by PF and CF is unaltered in the absence of NeuroD2.

*Neurod2* expression is maintained in granule cells during adult stages^38,39^. Thus, we next addressed the long-term survival of granule cells and their stable integration into the cerebellar circuit in adult *Neurod2*^−/−^ mutants. Immunostaining for calretinin and vesicular glutamate transporter 1 (VGLUT1) to label granule cell axons and their terminals, respectively, and PV to mark Purkinje cell dendrites, showed unaltered PF trajectory and PF terminals along Purkinje cell dendrites in *Neurod2*^−/−^ mutants at 1 year of age (Fig. 3E; please note differences in interneuron-associated PV staining in the ML that are further addressed below). These findings are supported by electron microscopy. We observed normally structured nuclear heterochromatin and no signs of heterochromatic clumping (that precedes cell death) in granule cells from 1 year old *Neurod2*^−/−^ mutants (Fig. 3F). Moreover, electron microscopy revealed a similar density of PF-Purkinje cell spine synapses in *Neurod2*^−/−^ mutants (control, 37.8 +/− 4.8 synapses/100 µm^2^; mutant, 31.27 +/− 0.5 synapses/100 µm^2^; n = 3 each; p = 0.25), in line with the finding of largely unaltered granule cell and Purkinje cell densities in *Neurod2*^−/−^mutants. In summary, these results show that NeuroD2 is not essential for the morphological differentiation of granule cells and the long-term maintenance of glutamatergic inputs to the cerebellar cortex.

NeuroD1 is closely related to NeuroD2 and prominently expressed in granule cells suggesting that it functionally compensates for the loss of NeuroD2. Therefore, we generated *Neurod1/2* compound mutants by breeding *Neurod2*^−*/*−^ mutants to conditional *Neurod1* mutants^30^, in which recombination occurs in postmigratory granule cells using *GABA A*α*6*-Cre driver mice^40^. At three weeks of age we observed no granule cell apoptosis in conditional *Neurod1* null mutants with one wildtype copy of *Neurod2* (*Neurod2*^*+/*−^*; Neurod1*^*fl/*−^*; Cre*) or *Neurod2* null mutants with one functional copy of *Neurod1* (*Neurod2*^−*/*−^*; Neurod1*^*fl/*−^; Fig. 3G). In contrast, *Neurod1/2* double mutants (*Neurod2*^−*/*−^*; Neurod1*^*fl/*−^*; Cre*) were severely ataxic, and histological analysis revealed reduced cerebellar size and increased granule cell apoptosis at 3 weeks of age compared to single mutant controls (Fig. 3G). These findings identify highly overlapping survival functions of NeuroD1 and NeuroD2 in postmigratory granule cells.

### NeuroD2 promotes survival and terminal differentiation of ML interneurons

**Figure 4:**
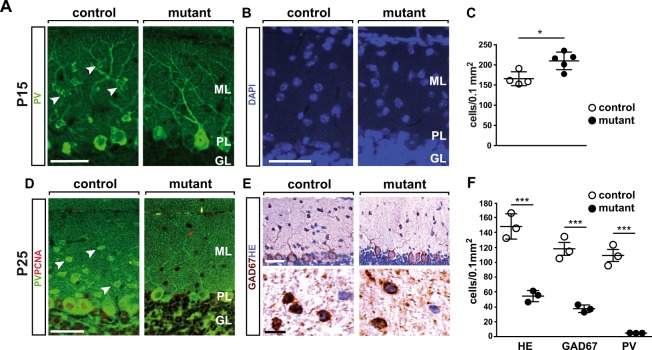
NeuroD2 is required for survival and terminal differentiation of MLIs. (**A**) Absence of PV^+^ MLI in the cerebellum of NeuroD1^−/−^ mutants. Fluorescent immunostaining of sagittal sections (lobule 5) at P15. Arrowheads point to PV^+^ MLIs in control mice. Scale bar, 50 µm. (**B**) Nuclear DAPI staining of sagittal sections (lobule 5) at P15. Scale bar, 50 µm. (**C**) Increased density of DAPI^+^ nuclei in the ML of *NeuroD*^−*/*−^ mutants. n = 4–5 per genotype. (**D**) Fluorescent immunostaining of sagittal sections (lobule 5) at P25. Arrowheads point to PV^+^ MLIs in control mice. Scale bar, 50 µm. (**E**) H&E staining and immunostaining for GAD67 on sagittal sections (P25, lobule 5). (**F**) Reduced density of GAD67^+^ interneurons in the ML of *NeuroD2*^−*/*−^ mutants. Quantification of H&E and GAD^+^ stainings in lobule 5, n = 3 per genotype.

In addition to glutamatergic granule cells, NeuroD2 is expressed by GABAergic MLIs^24^ (Fig. 1A), but its role in these cells after P25 is unknown. Immunostaining at both P15 and P25 demonstrated that PV-expressing MLIs were present in control mice, but virtually absent from the ML of *Neurod2*^−/−^ mutants (Fig. 4A,D). Next, we asked whether absence of PV marker expression resulted from a loss of MLI in *Neurod2*^−/−^ mutants. As determined by TUNEL staining, the number of apoptotic cells was not altered at P15 (data not shown), and cell density in the ML was even increased in *Neurod2*^−/−^ mutants (Fig. 4B,C). In stark contrast, hematoxylin and eosin (H&E) histology, as well as immunostaining for glutamate decarboxylase 1 (GAD67) demonstrated that MLI density was strongly reduced (to ~30%) in *Neurod2*^−/−^ mutants at P25 (Fig. 4E,F). Thus, we conclude that NeuroD2 is required for MLI survival at late postnatal stages. Since loss of PV expression preceded the reduction of MLIs, and the surviving population of GAD67^+^ MLIs lacked PV expression (Fig. 4F), we furthermore hypothesized that NeuroD2 acts during the terminal differentiation of these cells upstream of PV expression, but downstream of the expression of more basal GABAergic markers, such as GAD67.

**Figure 5:**
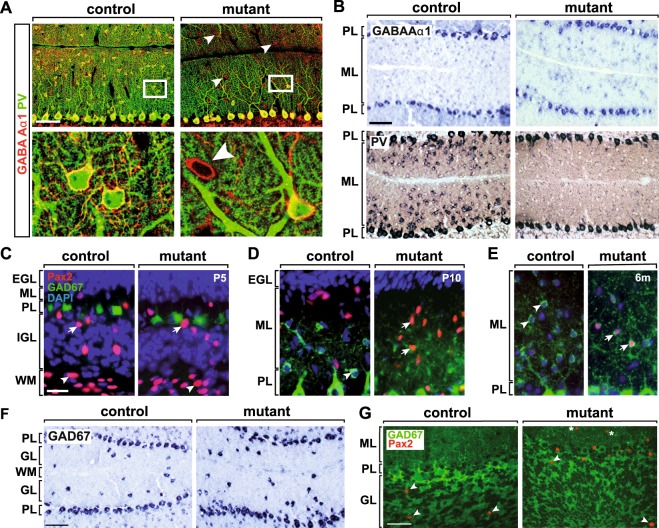
NeuroD2-deficient MLI are permanently arrested at a premature stage. (**A**) Surviving MLIs in *Neurod2*^−*/*−^ mutants express GABA Aα1 receptor (arrowheads), but only rarely PV. Fluorescent immunostaining of sagittal sections (lobule 5) at P25. Lower panels, magnification of boxed area in upper panels. Scale bars, 50 µm. (**B**) MLIs in *Neurod2*^−*/*−^ mutants are impaired at the transcriptional level to express PV, but not GABA Aα1 receptor. *In situ* hybridization at P17. Scale bar, 100 µm. (**C–E**) MLIs in adult *Neurod2*^−*/*−^ mutants maintain Pax2 expression. Fluorescent immunostaining for Pax2 and GAD67, and nuclear counterstaining (DAPI) on sagittal sections. *C*, Similar numbers of migrating Pax2^+^ interneuron precursors in the WM (arrowhead) and IGL (arrows) of *Neurod2*^−*/*−^ mutants and controls (P5). (**D**) Immature interneurons (P10) in controls settle in the ML and switch from Pax2 to GAD67 expression (arrowhead), following a gradient from the inner to the outer ML. MLIs in *Neurod2*^−*/*−^ mutants maintain Pax2 expression and show delayed onset of GAD67 expression (arrows). (**E**) No detectable Pax2 expression in control MLIs (arrowheads) at 6 months. In contrast, persistent co-expression of Pax2 and GAD67 (arrows) in MLIs of *Neurod2*^−*/*−^ mutants. Scale bar, 20 µm. (**F**) Golgi interneurons terminally differentiate in the GL of *Neurod2*^−*/*−^ mutants. *In situ* hybridization for GAD67 (P17). Scale bar, 100 µm. (**G**) Maintenance of Golgi cells (arrowheads) in adult *Neurod2*^−*/*−^ mutants. Fluorescent immunostaining for GAD67 and Pax2 (age 6 months). Dashed lines mark the PL (note absence of GAD67^+^ basket cell terminals in the PL of *Neurod2*^−*/*−^ mutants). Asterisks, persistent Pax2 expression in MLIs of *Neurod2*^−*/*−^ mutants. Scalebar, 50 µm. EGL, external granule cell layer; GL, granule cell layer; IGL, internal granule cell layer; ML, molecular layer; PL, Purkinje cell layer; WM, white matter.

To further test this hypothesis, we performed additional immunostainings at P25 and found that the remaining population of GAD67^+^/Parv^−^ MLIs in *Neurod2*^−/−^ mutants maintained expression of the GABA Aα1 receptor (Fig. 5A). We verified these findings by *in situ* hybridization and found persistent expression of tha GABA Aα1 receptor (Fig. 5B), GAD67, and the vesicular GABA transporter (VGAT) (data not shown) in surviving MLIs, but a dramatic loss of *PV* transcripts in *Neurod2*^−/−^ mutants (Fig. 5B). Finally, we examined the expression of Pax2, a transcription factor that marks the transition from proliferative precursor cells to terminally differentiating postmitotic MLIs^41,42^. At P5, we observed a similar number and distribution of Pax2-positive interneuron precursors in the prospective white matter (lobule 5; control, 358.3 +/− 32.7, n = 4 sections from 2 mutants; mutant, 391.4 +/− 24.22, n = 6 sections from 3 mutants; p = 0.54), IGL, and ML of *Neurod2*^−/−^ mutants when compared to controls (Fig. 5C), suggesting that NeuroD2 is not required for the production and initial migration of interneuron precursors. At P10, the loss of Pax2 expression in the ML of controls (following an inside-out gradient) was paralleled by GAD67 accumulation. In contrast, Pax2 expression was maintained and the acquisition of GAD67 immunoreactivity delayed in the ML of *Neurod2*^−/−^ mutants (Fig. 5D). Pax2 was completely absent from MLIs in adult controls, but persisted in the majority of NeuroD2-deficient MLIs at P50 (data not shown) and even at 6 months of age (Fig. 5E). This suggests that surviving mutant MLIs retain an immature developmental state. By contrast, we found no evidence that Golgi cells, the GABAergic interneurons of the GL, were affected in *Neurod2*^−*/*−^ mutants. *In situ* hybridization for GAD67 (Fig. 5F) and VGAT (not shown) at P17 demonstrated timely differentiation of Golgi cells in the GL of *Neurod2*^−*/*−^ mutants. Quantification revealed a tendency towards a reduced total number of GAD67-immunolabeled Golgi cells in the GL of lobule 5 (control, 35.67 +/− 2.8; mutant, 25.33 +/− 2.96, p = 0.0644; n = 3 each). However, when normalized to GL area, Golgi cell density was not changed (control, 69.59 +/− 9.55 cells/mm²; mutant, 69.38 +/− 7.42 cells/mm², n = 3 each; p = 0.987), similar to our findings for granule cells and Purkinje cells. In contrast to MLIs, mature Golgi cells maintain Pax2 expression during adult stages. Immunostaining for Pax2 identified a normal number of Pax2^+^ Golgi cells in the GCL of *Neurod2*^−*/*−^ mutants compared to controls at 6 months of age (Fig. 5G). Since Golgi cells provide both feedforward and feedback inhibition to granule cells^43^, these findings further support an intact functional integration of granule cells into the cerebellar network. In summary, we posit that NeuroD2 is not necessary to instruct a GABAergic phenotype in surviving MLIs, but is required for terminal differentiation of GABAergic interneurons in the ML of the cerebellum.

### Irregular basket cell axon growth, impaired formation of basket cell terminals, and decreased synaptic inhibition of Purkinje cells in *Neurod2*^−/−^ mutants

**Figure 6:**
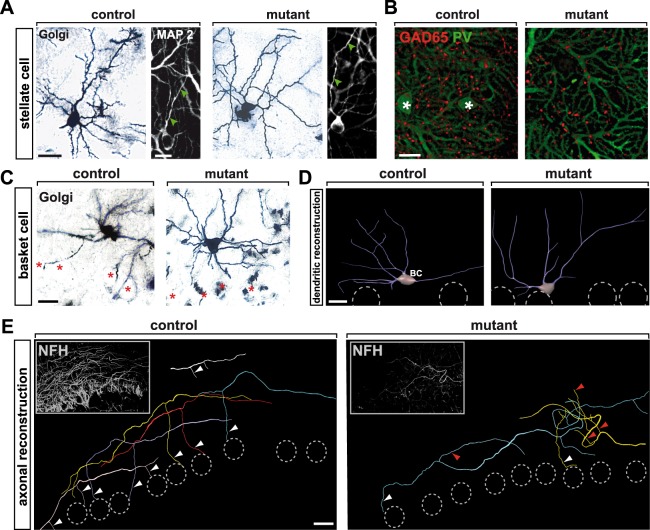
NeuroD2 controls directed growth of basket cell axons. (**A**) Unaffected growth and arborisation of stellate cell dendrites in *Neurod2*^−*/*−^ mutants. Golgi silver impregnation of cryosections (60 µm) at P25. Confocal analysis of fluorescent immunostainings for MAP2 at P25. Arrowheads point to dendritic profiles. Scale bars, 20 µm (left); 10 µm (right). (**B**) GABAergic terminals (presumably derived from stellate cells) that synapse on Purkinje cell dendrites in the outer ML of *Neurod2*^−*/*−^ mutants. Confocal micrographs of fluorescent immunostainings for GAD65 (stellate cell terminals) and PV (Purkinje cell dendrites) at P25. Asterisks mark stellate cell bodies in controls. Scale bar, 10 µm. (**C**,**D**) Normal development of basket cell dendrites in the inner third of the ML from *Neurod2*^−*/*−^ mutants compared to controls. (**C**) Golgi silver impregnation of cryosections (60 µm) at P25. Asterisks mark Purkinje cell somata. Scale bar, 20 µm. (**D**) Morphometric 3D reconstruction of dendrites at P25 marked by MAP2 labeling. Dashed circles indicate Purkinje cell somata. BC, basket cell. Scale bars, 20 µm. (**E**) Morphometric 3D reconstruction of basket cell axons after immunostaining for NFH at P25. Shown are five (control) and three (*Neurod2*^−*/*−^ mutants) axons (color coded) corresponding to distinct basket cells. In control mice axons extend collaterals (arrowheads) towards Purkinje cell somata (dashed circles). *Neurod2*^−*/*−^ mutant axons follow random trajectories (red arrowheads) and only rarely contact Purkinje cells (white arrowhead). Insets show merged confocal stacks used for reconstruction. Note reduced NFH staining in *Neurod2*^−*/*−^ mutants due to reduced number of basket cells. Scale bar, 20 µm.

To elucidate specific NeuroD2 controlled functions in MLI development, we analyzed morphological differentiation of MLIs in more detail. Golgi staining and immunostaining for the dendritically expressed microtubule-associated protein MAP2 at P25 indicated that stellate cell morphology, including dendritic arborization in the outer ML, was not grossly altered in *Neurod2*^−/−^ mutants (Fig. 6A). Immunostaining for GAD65 and PV in the upper ML, where most presynaptic terminals are derived from stellate cells^11,12^, showed GABAergic puncta aligned along Purkinje cell dendrites (Fig. 6B). Together, these data suggest that surviving stellate cells in *Neurod2*^−/−^ mutants develop dendrites, axons, and synapses onto Purkinje cells. However, the unequivocal identification and quantification of stellate cell/Purkinje cell synapses will require further studies, including higher-magnification microscopic images of pre- and postsynaptic structures.

As in the case of stellate cells, mutant basket cells in the inner ML developed normal dendritic arbors, as revealed by Golgi silver staining (Fig. 6C) and 3D reconstruction of MAP2 labeled dendrites at P25 (Fig. 6D) (total dendritic length: control, 351 ± 18 µm, n = 20 cells; mutant, 421.8 ± 31.3 µm; n = 30 cells; p = 0.09). To determine their growth trajectories, we labeled basket cell axons at P25 by fluorescent immunostaining for the neurofilament heavy chain (NFH) and performed 3D reconstructions of individual axons (Fig. 6E). As expected from the lower number of basket cells, the number of NFH labeled basket cell axons was prominently reduced in the ML of *Neurod2*^−/−^ mutants. Strikingly, while axons in control mice grew along the translobular plane and extended collaterals towards the PL, basket cell axons in *Neurod2*^−/−^ mutants followed random trajectories, even towards the pial surface. Consequently, very few of the basket cell axon collaterals descended towards the PL in *Neurod2*^−/−^ mutants to form contacts with Purkinje cells (Fig. 6E).

**Figure 7:**
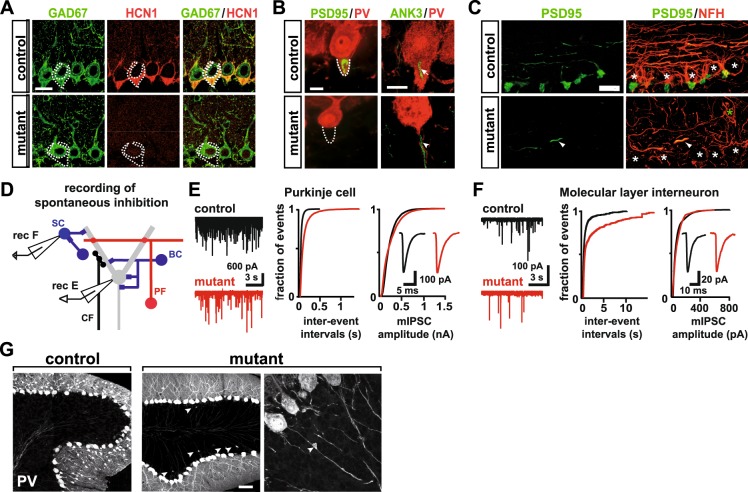
Absence of inhibitory basket cell terminals and Purkinje cell axon pathology in *Neurod2*^−*/*−^ mutants. (**A,B**), Absence of basket cell terminals at the soma (pericellular basket) and the AIS (pinceau formation) of Purkinje cells (dashed areas in **A, B**) in *Neurod2*^−*/*−^ mutants. Fluorescent immunostaining for (**A**) GAD67/HCN1 and (**B**) PSD95/PV; ANK3/PV (P25). Note that HCN1 and PSD95 mark basket cell terminals, whereas GAD67 and PV label both basket cell terminals and Purkinje cells. Immunostaining for the AIS marker ANK3 indicates unaltered AIS assembly in Purkinje cells of *Neurod2*^−*/*−^ mutants (arrowhead) compared to controls (arrow). Scale bar, 20 µm (**A**), 10 µm (**B**). (**C**) Targeted basket cell axon growth and formation of inhibitory presynaptic terminals is permanently abolished in *Neurod2*^−*/*−^ mutants. Confocal analysis of fluorescent immunostaining for PSD95 and NFH at P75. Basket cell axons are reduced in number and show undirected trajectories (asterisks mark Purkinje cell somata). Scale bar, 20 µm. (**D**) Schematic of electrophysiological recordings of mIPSCs in Purkinje cells and MLIs. (**E**) Left: Representative traces of Purkinje cell mIPSC recordings reveal reduced mIPSC frequency in *Neurod2*^−*/*−^ mutants (red, bottom) compared to control (black, top). Middle: Cumulative probability histogram indicates longer mIPSC inter-event intervals for *Neurod2*^−*/*−^ mutants. Right: Cumulative probability histogram of control (n = 16 cells) and mutant Purkinje cell mIPSCs (n = 15 cells) indicates similar mIPSC amplitude distributions in *Neurod2*^−*/*−^ mutants and controls. Inset, average mIPSCs of control (black) and *Neurod2*^−*/*−^ mutant (red). (**F**) Left: Representative traces of MLI mIPSC recordings show reduced mIPSC frequency in *Neurod2*^−*/*−^ mutants (red, bottom) compared to control (black, top). Middle: Cumulative probability histogram indicates longer mIPSC inter-event intervals for *Neurod2*^−*/*−^ mutants compared to control. Right: Cumulative probability histogram of control (n = 8 cells) and mutant MLI mIPSCs (n = 9 cells) indicates similar mIPSC amplitude distributions in controls and *Neurod2*^−*/*−^ mutants. Inset, average MLI mIPSCs of control (black) and *Neurod2*^−*/*−^ mutant (red). (**G**) Axonal swellings proximal to the AIS in Purkinje cells of *Neurod2*^−*/*−^ mutants. Fluorescent immunostaining for PV at P25. Scale bar, 50 µm.

To test whether aberrant basket cell axon growth in *Neurod2*^−/−^ mutants compromises the formation of basket cell terminals at Purkinje cells, including the characteristic basket cell pinceau at the axon initial segment (AIS), we performed immunostainings for two specific markers: the hyperpolarization activated cyclic nucleotide-gated potassium channel 1 HCN1^44^ and postsynaptic density protein 95 (PSD95)^45^. Both markers were absent from the perisomatic compartment and AIS of Purkinje cells in *Neurod2*^−/−^ mutants at P25 (Fig. 7A,B). Since postsynaptic targeting of ankyrin 3 (ANK3) to the AIS is required for the pinceau assembly^46^, its presence in Purkinje cells of *Neurod2*^−/−^ mutants (Fig. 7B) indicates normal AIS formation and suggests that disrupted pinceau formation is a basket cell-intrinsic defect. Furthermore, immunostaining for NFH and PSD95 at P75 demonstrated that PL-directed basket cell axon growth was permanently disrupted and not only developmentally delayed in *Neurod2*^−/−^ mutants (Fig. 7C). We conclude that NeuroD2 controls a basket cell-intrinsic transcriptional program that regulates targeted axon growth and presynaptic differentiation, but is not essential for MLI dendritogenesis.

We next addressed whether the reduction in the number of MLIs and the morphologically abnormal basket cell axons result in decreased synaptic inhibition to Purkinje cells. To test whether the number of functional inhibitory synapses is diminished in *Neurod2*^−/−^ mutants, we examined the frequency of miniature inhibitory postsynaptic currents (mIPSCs) in Purkinje cells of acute cerebellar slices (Fig. 7D). In the presence of sodium channel, glutamate receptor, GABA B receptor, and glycine receptor blockers, we found that mIPSC frequency was significantly reduced in *Neurod2*^−*/*−^ mutants (Fig. 7E; left: control, 26.9 ± 1.7 Hz, n = 15; mutant, 15.4 ± 1.4 Hz, n = 16, p < 0.0001; middle: cumulative probability histogram of inter-event intervals, p < 0.0001, Kolmogorov-Smirnov test), whereas mIPSC amplitude was unaffected (Fig. 7E; right: control, 225.5 ± 9.0 pA, n = 15; mutant, 230.8±17.3 pA, n = 16, p = 0.8; cumulative probability histogram, p = 1.0, Kolmogorov-Smirnov test), as well as mIPSC kinetics (control rise time 0.47 ± 0.07 ms, n = 8, mutant rise time, 0.46 ± 0.06 ms, n = 7, p = 0.43, t-test; control decay time 4.0 ± 0.4 ms, n = 8, mutant decay time, 3.5 ± 0.2 ms, n = 7, p = 0.1, t-test), suggesting no major differences in synaptic release of GABA from remaining MLIs. However, we found that input resistance (Ri) of Purkinje cells was increased in *Neurod2*^−*/*−^ mutants (control, 23.6 ± 3.8 MOhm, n = 8; mutant, 51.1 ± 8.5 MOhm, n = 7, p < 0.05), consistent with decreased GABAergic input^47^. To test whether the differences in Ri between control and mutant are due to intrinsic Purkinje cell properties or GABAergic input, we recorded spontaneous Purkinje cell firing in the presence of synaptic blockers. The average firing frequencies of Purkinje cells were similar in mutants and controls and thus suggest that Purkinje cell excitability was not grossly affected by NeuroD2 deletion (control: 30.8 +/− 4.1 Hz, n = 12, mutant: 27.3 +/− 3.0 Hz, n = 17, p = 0.49; see below). Together, these data indicate that GABAergic inputs onto Purkinje cells are reduced in number, but that GABAergic transmission at remaining inputs is intact in *Neurod2*^−*/*−^ mutants. MLIs also form reciprocal GABAergic synapses^48^. We therefore tested whether inhibition was also impaired in MLIs of *Neurod2*^−*/*−^ mutants. To preferentially target stellate cells, MLIs from the outer two thirds of the ML were recorded (Fig. 7D). Indeed, mIPSC frequency was decreased in MLIs from *Neurod2*^−*/*−^ mutants compared to controls (Fig. 7F; left: control, 3.3 ± 1.2 Hz, n = 8; mutant, 0.5 ± 0.2 Hz, n = 9; p < 0.05; middle: cumulative probability histogram of inter-event intervals, p < 0.0001, Kolmogorov-Smirnov test), whereas mIPSC amplitudes were similar (Fig. 7F; right: control, 80.7 ± 18.9 pA, n = 9; mutant, 86.7 ± 16.1, n = 8, p = 0.82; cumulative probability histogram, p = 1.0, Kolmorogov-Smirnov test). Thus, remaining MLIs receive reduced inhibitory input, in line with the postnatal loss of MLIs in *Neurod2*^−*/*−^ mutants. Because inhibitory input to MLIs is sparse, counter to Purkinje cells, we did not observe a difference in Ri between control and mutant MLIs (control, 426.6 ± 97.0 MΩ, n = 6; mutant, 504 ± 185 MΩ, n = 5, p = 0.7, t-test), suggesting that decreased inhibitory input onto mutant MLIs does not alter intrinsic electrical properties of surviving MLIs or affect MLI excitability.

Consistent with intact glutamatergic input and unaltered mIPSC amplitudes of the remaining GABAergic inputs, we observed no morphological abnormalities in the somatodendritic compartment of Purkinje cells in *Neurod2*^−*/*−^ mutants (Figs 3E,F and 5A). In addition, somatic recordings of spontaneous firing rates in Purkinje cells in the presence of synaptic blockers showed no differences between controls and *Neurod2*^−*/*−^ mutants (control, 30.8 ± 4.0 Hz, n = 12; mutant, 27.3 ± 3.0 Hz, n = 17; p = 0.49). This indicates that action potential generation is not compromised and further suggests that the somatodendritic compartment of Purkinje cells in *Neurod2*^−*/*−^ mutants is normal. Nevertheless, the identification of minor structural and functional changes in Purkinje cell dendrites of *Neurod2*^−*/*−^ mutants will require further investigation. In contrast to dendrites, we frequently observed swellings of Purkinje cell axons proximal to the (basket cell terminal-lacking) AIS in *Neurod2*^−*/*−^ mutants (Fig. 7G). In conclusion, loss of basket cell contacts has no major effects on the somatodendritic compartment of Purkinje cells, but could result in axonal pathology.

**Figure 8:**
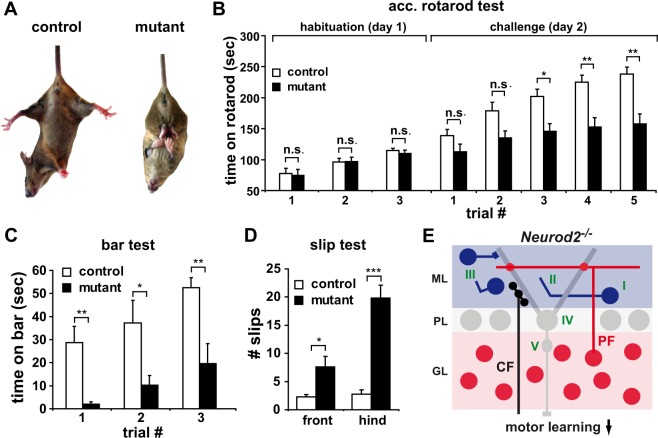
Complex motor functions, but not basic locomotor activity, are impaired in *Neurod2*^−*/*−^ mutants. (**A**) A majority of *Neurod2*^−*/*−^ mutants display hind limb clasping. (**B**) Normal basic motor performance in *Neurod2*^−*/*−^ mutants (age 5 months) on a rotarod at low acceleration rate (three consecutive tests, acceleration rate 3.3 to 12 rpm within 2 min). At higher acceleration rates (4.1 to 41 rpm within 6 min), *Neurod2*^−*/*−^ mutants exhibit a motor learning deficit (control, n = 27–32; mutant, n = 23–24). (**C**) Reduced balance performance of *Neurod2*^−*/*−^ mutants during the bar test (bar, 1.8 cm in diameter; three consecutive trials; control, n = 6; mutant, n = 8). (**D**) *Neurod2*^−/−^ mutants show an increased number of front and hind limb slips in the slip test (three min exploration time; 30 × 30 cm elevated grid; control, n = 4; mutant, n = 5). (**E**) Model of cerebellar network deficits in *Neurod2*^−*/*−^ mutants. NeuroD2 shares redundant functions with NeuroD1 in postmigratory granule cells (red), but is not essential for normal excitatory input onto Purkinje cells (grey), provided by PF and CF. In contrast, NeuroD2 serves non-redundant functions during survival and maturation of MLIs (blue) (I), in directed growth of basket cell axons and the formation of inhibitory presynaptic terminals at Purkinje cells (II), and stellate cells (III). This causes a reduction of inhibitory neurotransmission in Purkinje cells (IV) (shifting the ratio of excitatory/inhibitory neurotransmission towards increased excitation) and results in swellings of Purkinje cell axons (V). Together, these deficits in inhibitory circuits likely affect Purkinje cell output to deep cerebellar nuclei and may impair advanced motor learning.

Purkinje cell output coordinates motor functions. Locomotor activity of unchallenged *Neurod2*^−/−^ *mutants* was not different from wildtype littermates (data not shown), but a majority of *Neurod2*^−/−^ mutants showed hind limb clasping upon tail suspension (Fig. 8A). *Neurod2*^−/−^ mutants showed normal endurance on a rotarod at constant speed (controls, 42.02 ± 2.58 min, n = 4; mutants, 42.39 ± 2.33 min; n = 5; p = 0.93), and performed normally when trained on an accelerated rotarod in three consecutive trials at a low acceleration rate (3.3 to 12 rpm within 2 min; 10 min intertrial intervals), (Fig. 8B; ‘habituation’ trial 1, controls, 74.8 ± 9.5 s; mutants, 77.4 ± 8.4 s; p = 0.97; trial 2, controls, 96.3 ± 5.9 s; mutants, 97.0 ± 6.7 s, p = 0.97; trial 3, controls, 114.7 ± 3.9 s; mutants, 109.9 ± 5.4 s, p = 0.85; control, n = 32; mutant, n = 24). In contrast, when tested for 5 consecutive trials using a higher acceleration rate (4.1 to 41 rpm within 6 min; 10 min intertrial intervals), the performance of *Neurod2*^−/−^ *mutants* was reduced compared with controls (Fig. 8B; ‘challenge’; trial 1, controls, 138.7 ± 10.1 s; mutants, 112.4 ± 2.7 s, p = 0.36; trial 2, controls, 178.8 ± 13.7 s, mutants, 134.8 ± 11.6 s, p = 0.09; trial 3, controls, 201.7 ± 2.3 s, mutants, 145.4 ± 12.8 s, p < 0.02; trial 4, controls, 227.5 ± 11.5 s, mutants, 146.0 ± 14.0 s, p < 0.01; trial 5, controls, 240.1 ± 11.6 s mutants, 151 ± 15.6, p < 0.01; controls, n = 27; mutants, n = 23). Furthermore, the ability of *Neurod2*^−/−^ mutants to balance on a bar (1.8 cm in diameter) was strongly impaired and improved less compared to controls when tested in 3 consecutive trials with 10 min intertrial intervals (Fig. 8C; trial 1, controls, 28.7 ± 7.8 s; mutants, 2.0 ± 0.9; p < 0.01; trial 2, controls, 37.2 ± 10.8 s, mutants, 10.3 ± 3.9 s, p < 0.05; trial 3 controls, 52.5 ± 4.8 s, mutants, 19.6 ± 7.9 s, p < 0.05; controls, n = 6; mutants, n = 8). Finally, when freely moving on a grid (30 × 30 cm, 1.5 cm distance between stacks, 3 min), *Neurod2*^−/−^ mutants showed significantly more front limb and hind limb slips (Fig. 8D; front limb, controls, 2.3 ± 0.5 slips; mutants, 8.4 ± 1.8 slips, p < 0.05; hind limb, controls, 2.8 ± 0.9 slips, mutants, 19.8 ± 2.6 slips, p < 0.0007; controls, n = 4; mutants, n = 5). We conclude that challenging motor activity, such as balancing and advanced motor learning, but not basic locomotion, is impaired in *Neurod2*^−/−^ mutants. In conclusion, we suggest a working model according to which the loss of inhibitory contacts provided by MLIs results in a shifted excitatory/inhibitory input ratio in Purkinje cells and compromises the integrity of Purkinje cell axons. Impaired inhibitory Purkinje cell output may impair adaptive motor learning (Fig. 8E).

## Discussion

NeuroD2 deficiency in a mixed C57Bl/6 × 129/Sv background is associated with reduced serum TH levels^33^, severe growth retardation and postnatal death^24^. Genetic background heavily affects phenotype expressivity in many mouse mutants^49^. Accordingly, backcrossing of our *Neurod2* mutants to a 129/SvJ background restored normal blood TH levels and viability, and allowed us to examine NeuroD2 functions in the cerebellar cortex in the absence of confounding TH deficits. We found that ~70% of granule cells are preserved in adult *Neurod2*^−/−^ mutants, demonstrating that NeuroD2 is required for granule cell survival only during a critical postnatal period (until ~P20). Timely and complete CF elimination as well as normal short-term plasticity of PF and CF inputs in Purkinje cells of *Neurod2*^−/−^ mutants indicated that mutant granule cells structurally and functionally integrate into the cerebellar circuit. Thus, our findings suggest that NeuroD2 is not essential for the formation and functional integration of glutamatergic inputs in the cerebellum. NeuroD1 and NeuroD2 are co-expressed in granule cells^39^. Granule cell apoptosis was strikingly increased in *Neurod1/2* double mutants, in which *Neurod1* disruption occurs in postmigratory granule cells. We conclude that NeuroD1 and NeuroD2 serve redundant survival functions in postmigratory granule cells.

We observed markedly reduced MLI numbers (~30% of controls) in *Neurod2*^−/−^ mutants at P25, but not at P15. This suggests that NeuroD2 is required for MLI survival during a critical postnatal period, i.e. after their arrival in the nascent ML. Loss of MLIs may result from a failure to properly execute a terminal differentiation program^50^. In support of this hypothesis, surviving NeuroD2-deficient MLIs maintain Pax2 expression, a marker of immature interneurons^41,42^. Concomitantly, mutant MLIs lack expression of differentiation markers, such as PV and HCN1 ion channels. We speculate that these factors are integral components of a NeuroD2-controlled MLI terminal differentiation program. However, whether *Pvalb* and *Hcn1* are bona fide NeuroD2 target genes requires further investigation. The abnormal expression status of MLIs is associated with a specific impairment in the morphological differentiation of basket cells, as basket cell axon branching directed to the PL and inhibitory terminal formation, but not dendritic growth, are severely affected in *Neurod2*^−/−^ mutants. Irregular basket cell axon growth could be the consequence of detrimental effects of persistent Pax2 expression on basket cell differentiation. A previous study indicates that continuous Pax2 expression in the renal epithelium of transgenic mice causes kidney abnormalities, possibly by instilling an immature state^51^. Furthermore, NeuroD2 deficiency may impair the expression of axonal signaling receptors, similar to the situation in *Neurod2/6* double mutants, in which disrupted callosal axon tract formation is associated with the loss of contactin-2 expression^28^. A candidate receptor in basket cell axons is Neuropilin-1, which mediates SEMA3A-induced axon guidance signaling during basket cell axon branching^52^.

Consistent with a significant reduction in MLI number and undirected basket cell axon growth, we found that inhibitory terminals are diminished in the ML of *Neurod2*^−/−^ mutants. Although the density of GABAergic synapses is reduced throughout the entire ML, the most striking consequence of *Neurod2* deletion remains the absence of functional GABAergic basket cell terminals. A previous study suggested that basket cells mediate large-amplitude calcium store-dependent mIPSCs in Purkinje cells^53^. However, mIPSC amplitude distributions of control and *Neurod2*^−*/*−^ mutants were similar, indicating that stellate cells may also generate such large mIPSCs. In addition to intact excitation, CF elimination from Purkinje cell dendrites also requires GABAergic inhibition^54^. Despite the finding that mIPSC frequency is decreased by >50% in *Neurod2*^−*/*−^ mutants, we found that CFs are pruned by P19 in *Neurod2*^−*/*−^ mutants. We conclude that residual Purkinje cell inhibition by stellate cells (and possibly Purkinje cell collaterals) is sufficient for the efficient elimination of CF synapses in *Neurod2*^−*/*−^ mutants.

Our data indicate that Purkinje cell dendrites and the AIS develop properly in the absence of perisomatic basket cell innervation. Since they appeared anatomically normal, intrinsic functional properties of Purkinje cells were not rigorously tested in electrophysiological experiments. To clarify this issue in detail, more experiments in current clamp would be required, e.g. step current injections with varying amplitudes. However, spontaneous firing frequencies of PCs measured in the presence of synaptic blockers were similar, suggesting that intrinsic properties of PCs were not dramatically altered in mutants. Input resistance in mutants was assessed with GABAergic transmission intact. The apparent increase in input resistance in mutants is consistent with decreased inhibition, as previously shown^55^. Futher experiments could test how partial loss of inhibtion in the cerbellar cortex alters its output, e.g. *in vivo* recordings from deep cerebellar nuclei (DCN).

Our data suggest that basket cell terminals could serve a role in maintaining axonal integrity as we frequently observed swellings of Purkinje cell axons proximal to the AIS in *Neurod2*^−/−^ mutants. Axonal swellings (‘torpedoes’) are a common feature of various neurodegenerative diseases with cerebellar involvement, but also occur in the developing mouse cerebellum and peak at P11^56,57^. Axonal swellings in *Neurod2*^−/−^ mutants are not merely a consequence of abolished inhibition, as the removal of GABA A receptor-mediated synaptic inhibition in Purkinje cells causes no corresponding axonal pathology^50^. Instead, we suggest that the formation of an intact pinceau is required for maintaining the long-term integrity of Purkinje cell axons. Interestingly, it has recently been questioned whether the pinceau serves GABAergic signaling at all^51^. Instead, it may generate high resistivity in the AIS environment, serving the electrical inhibition of the proximal axon^52^. We thus speculate that the pinceau assumes an axoprotective function by controlling AIS excitability, and despite normal spontaneous firing rates in the Purkinje cell soma, it remains to be determined whether action potential propagation along the Purkinje cell axons is affected in *Neurod2*^−*/*−^ mutants.

Purkinje cells control motor behavior via inhibitory projections to deep cerebellar and vestibular nuclei^58^. *Neurod2*^−*/*−^ mutants showed normal basic locomotor behavior but impaired balancing and advanced motor learning, similar to the genetic blockade of all synaptic inhibition in Purkinje cells, which affects vestibulo-cerebellar motor learning, but not baseline motor performance^59^. Feed-forward inhibition by MLIs temporally modulates rate and regularity of Purkinje cell firing^15,16,18,19,58^. Thus, MLI-mediated inhibition of Purkinje cells may play an important role in motor learning. However, due to the global nature of the mutation, we cannot exclude that changes in granule cells or other brain regions, e.g. motor cortex, DCN, and basal ganglia, contribute to or even underlie motor learning deficits. However, we hypothesize that diminished inhibition of Purkinje cells in *Neurod2*^−/−^ mutants together with normal PF- and CF-mediated excitation results in an imbalance of excitation and inhibition in Purkinje cells, ultimately contributing to altered Purkinje cell firing and deficits in motor learning.

## Materials and Methods

### Mouse mutants

For routine genotyping genomic DNA was isolated from tail biopsies (Invisorb Spin Tissue Mini Kit, Invitek). *Neurod2* mutants were genotyped with primers #1 (Neurod2-s, 5′-TGG GCT CTC TCG GAG ATC T-3′), #2 (revNeo-s, 5′-ATT GTC TGT TGT GCC CAG TC-3′) and #3 (Neurod2-as, 5′-CTG TTG GGA GGT GGG GAG AG-3′). Conditional *Neurod1* mutants were genotyped as described^30^. Mice were bred and kept in the animal facility of the Max Planck Institute of Experimental Medicine with a 12 h light/dark cycle and 2–4 animals per cage. All animal experiments were performed in compliance with the regulations of the German Federal State of Lower Saxony for the use of experimental animals as approved by the “Niedersächsisches Landesamt für Verbraucherschutz und Lebensmittelsicherheit” (LAVES, Oldenburg)

### RNA analysis

Cerebellar tissue was stored in RNAlater (Ambion) at 4 °C. Total RNA was isolated using TRIzol reagent (Life Technologies) according to manufacturer’s instructions and purified using a RNeasy Mini kit (Qiagen). Concentration and integrity of purified RNA was confirmed using the Agilent 2100 Bioanalyser (Agilent Technologies). cDNA was synthesized using poly-thymidine and random nonamer primers and Superscript III RNase H reverse transcriptase (Invitrogen). Quantitative real-time PCR was carried out in triplicates with GoTaq qPCR Master Mix (Promega) on a 7500 Fast Real-Time PCR System (Applied Biosystems). Expression values were normalized to the housekeeping gene DNA topoisomerase type I (TOP1), and quantification was done by applying the ΔΔCt method, normalized to wildtype controls (set to 1). Primer sequences are available upon request.

### *In situ* hybridization

Mice were retrocardially perfused with 4% paraformaldehyde (PFA) in alkaline phosphate buffer (aPB, 0.1 M Na_2_HPO_4_ pH 9). Cerebella were dissected, fixed for 6 hours in 4% PFA in DEPC-treated PBS (DPBS), and washed with DPBS. Fixed tissue was submerged overnight in DPBS/25% sucrose and embedded in Tissue-Tek O.C.T.^TM^ Compound (Sakura). Tissue sections (16 µm) were cut with a cryomicrotome, collected on adhesive glass slides and stored at −80 °C. Preparation of digoxigenin-labeled RNA probes, hybridization to cryosections, and chromogenic staining was performed as described^28^. DNA templates for RNA probes were prepared using the following PCR primers: Gabra1-Fw (GABA Aα1 receptor): 5′-GAA AAA CAA CAC ATA TGC TCC TAC AG-3′; Gabra1-Rv (GABA Aα1 receptor): 5′-CTT ACC CTC TCT CTT CCT CTT GTC TA-3′; Parvalbumin-Fw: 5′-CCA GAG ACT TGT CTG CTA AAG AAA C-3′; Parvalbumin -Rv: 5′-ATT TTA TCA CAG CAA AGT CAA AAG C-3′; Gad67-Fw: 5′-GAG CGG ATC CTA ATA CTA CCA ACC T-3′; Gad67-Rv: 5′-AAC CAA TGA TAT CCA AAC CAG TAG AG-3′; VGAT-Fw: 5′-GTC GAG GGA GAC ATT CAT TAT CAG-3′; VGAT-Rv: 5′-GTA CAC AGC AGA CTG AAC TTG GAC-3′.

### Histology and Immunohistochemistry

Brain tissue preparation and immunohistochemistry were performed as described (Bormuth *et al*.^28^). Primary antibodies were directed against Ank3 (1:50, mouse IgG, Santa Cruz Biotechnology), Calretinin (Calb2; 1:1000, polyclonal rabbit; Millipore), HCN1 (1:200, polyclonal rabbit; Biomol), GABA Aα1 receptor (1:500, polyclonal rabbit, Millipore), GABA Aα6 receptor (1:500, polyclonal rabbit, Millipore), GAD67 (1:1000, mouse IgG, Millipore), GAD65 (1:1000, polyclonal rabbit, Millipore), GFAP (1:200 mouse IgG, Chemicon), MAP2 (1:800, mouse IgG, Millipore), NFH (1:200, polyclonal rabbit, Sigma), Parvalbumin (1:1000, mouse IgG, Sigma), Parvalbumin (1:000, polyclonal rabbit, Swant), Pax2 (1:250, polyclonal rabbit, Zymed), PCNA (1:300, polyclonal rabbit, Abcam), PSD95 (1:400, mouse IgG, Affinity Bioreagents), S100beta (1:200 monoclonal rabbit, Abcam), VGLUT1 (1:5000, polyclonal guinea pig, Millipore). Detection was performed with secondary antibodies conjugated to Alexa Fluor 488, 555 and 633 (1:1000, Thermo Fisher Scientific) and biotinylated secondary antibodies followed by diaminobenzidine (DAB; LSAB2 Kit, Dako; Vectastain Kit, Vector Laboratories).

### X-Gal staining

Free-floating cerebellar sagittal sections (20 µm) were collected in PBS in 24-well plates and incubated with 400 µl of X-Gal staining solution per well (5 mM K_3_[Fe(CN)_6_], 5 mM K4[Fe(CN)6], 2 mM MgCl2, 1.2 mg/ml 5-bromo-2-chloro-3-indoyl-beta-D galactopyranoside (X-gal) in PBS) at 37 °C. Stained sections were stored in PBS for subsequent immunostaining.

### TUNEL assay

Fragmented DNA of apoptotic cells in paraffin tissue sections was end-labeled using the DeadEnd colorimetric TUNEL system (Promega) according to manufacturer’s instructions.

### Golgi impregnation

Golgi impregnation of cryostat sections (50 µm) embedded with Eukitt (Sigma) was performed using a FD Rapid GolgiStain Kit (FD Neuro Technologies) according to manufacturer’s instructions.

### Electron microscopy

Mice were perfused with 4% PFA, 2.5% glutaraldehyde in 0.1 M phosphate buffer containing 0.5% NaCl. Sagittal cerebellar sections (1 mm) were contrasted with 2% osmium tetroxide (OsO_4_) in 0.1 M phosphate buffer and embedded in epoxy resin (Serva). Semithin sections (0.5 μm) were cut using an ultramicrotome (Leica) with diamond knife (Diatome). Sections were stained with azur II/methylenblue for 1 min at 60 °C or were de-eponized by using an etching solution according to Maxwell *et al*., 1978 followed by toluidine blue stain as described (Burns, 1978). Light-microscopic observation was performed by using (Leica DM RXA microscope). For electron microscopy, ultrathin sections (50–70 nm) were stained with 4% uranyl acetate followed by lead citrate and analyzed with a transmission electron microscope (EM109, Zeiss).

### Morphometric analysis

Digital images of chromogenic and fluorescent immunostainings were generated using a Zeiss Axiophot microscope, a Leica DM RXA microscope, and a LSM510 Meta Zeiss confocal laser-scanning microscope. Cerebellar size, lobule 3/5 area sizes, lobule 5 layer sizes, and MLI numbers (in cerebellar lobule 5) were quantified on sagittal sections (total cerebellar area: 2 sections/mouse; lobules 3/5: 6–7 sections/mouse; n = 3 per genotype) using ImageJ software. Apoptotic cells (as identified by TUNEL assay in the IGL) were quantified on 2 sagittal sections per mouse (n = 3). Granule cell density and MLI density was quantified on six toluidine blue stained 0.5 μm semithin sagittal sections per mouse in 0.02 mm^2^ regions of the dorsal GL in lobule 5 (n = 3 per genotype). Purkinje cell density was quantified on four toluidine blue and two methylen-blue stained 0.5 μm semithin sagittal sections per mouse in lobule 5 (n = 3 per genotype). PF to Purkinje cell synapses were identified by their typical ultrastructural morphology^60^, and their density was determined on 50 nm thick ultrathin sections in 100 μm^2^ regions of the ML in lobule 5. For the reconstruction of individual basket cells neurites, 12 μm deep z-stacks were collected in the translobular plain (40 images per stack) and analyzed with Neuromantic V.1.6.3 software for 3D reconstructions.

### Measurement of serum hormones

Blood was collected and serum prepared by centrifugation (10 min, 5000 rpm). Free (unbound) T_3_ and T_4_ serum levels were determined using a two-step chemiluminescent microparticle immunoassay (CMIA) and the Architekt i system (Abbott) following manufacturer’s instructions.

### Electrophysiology

Parasagittal or transverse cerebellar slices were prepared from *Neurod2*^−/−^ mutants and *Neurod2*^+/−^ littermate controls aged 14–19 days. Mice were decapitated, and the cerebellum was dissected out and glued to the stage of a vibroslicer (VT1200S, Leica Instruments), supported by a block of 4% agar. Dissection and cutting of slices was performed in ice-cold solution containing (in mM) 75 NaCl, 2.5 KCl, 0.5 CaCl_2_, 7 MgCl_2_, 1.25 NaH_2_PO_4_, 26 NaHCO_2_, 25 glucose, and 75 sucrose, bubbled with 95% O_2_–5% CO_2_. Slices of 300 μm thickness were cut and incubated at 34 °C for 30 min before use. During recordings, slices were superfused at a flow rate of 2–3 ml/minute with a solution containing (in mM) 125 NaCl, 2.5 KCl, 2.5 CaCl_2_, 1.3 MgCl_2_, 26.2 NaHCO_2_, 1 NaH_2_PO_4_, and 11 glucose with the addition of 100 μM picrotoxin to record excitatory postsynaptic currents (EPSCs), or 5 µM 2,3-dihydroxy-6-nitro-7-sulfamoyl-benzo[f]quinoxaline-2,3-dione (NBQX) to record IPSCs. 2 µM CGP, 1 µm strychnine, 50 µM APV were added to block GABA B, glycine and NMDA receptors, respectively. To minimize series resistance (Rs) errors, CF-EPSCs were recorded in the presence of 600–800 nM NBQX. Tetrodotoxin (TTX; 500 nM) was included in the bath solution to record miniature IPSCs (mIPSCs). Drugs were purchased from Tocris Bioscience (Ellisville, MO), Abcam (Cambridge, MA) or Sigma (St. Louis, MO).

Whole-cell recordings were made from optically identified cells with a gradient contrast system (Dodt *et al*., 2002) using a 60x water-immersion objective on an upright microscope (Olympus BX51WI). Pipettes for Purkinje cell-EPSC recordings were pulled from leaded glass capillaries (WPI, Sarasota, FL) with resistances of 1.0 to 1.5 MΩ, pipettes of 3–4 MOhm were used for MLI recordings. For Purkinje cell recordings, Rs as measured by the instantaneous current response to a 2 mV step with only the pipette capacitance canceled, was always less than 5 MΩ (usually less than 3 MΩ) and Rs was routinely compensated at 80%. Rs in MLI recordings was <25 MΩ and was not compensated. Pipette solutions for EPSC recordings contained (in mM) 35 CsF, 100 CsCl, 10 EGTA, and 10 HEPES, 0.3 mM D600 and 5 mM QX314, adjusted to pH 7.2 with CsOH. mIPSCs from Purkinje cells and MLIs in the distal two thirds of the ML were recorded at a holding potential of -70 mV with pipettes containing a solution (in mM) 150 CsCl, 2 MgCl_2_, 0.1 CaCl_2_, 1 EGTA, 10 HEPES, 0.4 Na-GTP, 4 Mg-ATP, adjusted to pH 7.3 with CsOH.

CFs or PFs were stimulated with brief voltage pulses (2–10 V, 20–200 μs) using theta glass pipette filled with extracellular solution placed in the GL or ML, respectively. Currents were recorded with a MultiClamp 700B amplifier (Axon Instruments, Sunnyvale, CA), filtered at 4 kHz and digitized (Digidata 1440 A, Axon Instruments) at 20–50 kHz using Clampex acquisition software. All experiments were performed at 30°-33 °C attained with an in-line heating device (Warner Instruments, Hamden, CT). Data was analyzed using AxographX (Sydney, Australia), Excel (Microsoft, Redmond, WA) and Prism (Graphpad, La Jolla, CA). For EPSC and IPSC kinetics, 20–80% rise time and weighted decay time are reported. To calculate weighted decay time the EPSCs or IPSCs were normalized to the peak, and the area from peak to baseline was integrated. For detection of mIPSCs a variable amplitude sliding template was used. Detection threshold was 2–3 SDs from noise, and detected events were visually inspected after detection for accuracy. Corrupt events were rejected from further analysis. Although clustered events were uncommon (with the exception for mIPSC recordings in control Purkinje cells), the first event in a cluster was accepted for events that were separated less than 5 ms, all events that were more than 5 ms apart were included in the analysis.

### Behavioral analysis

Motor function was investigated on a rotating rod (diameter 3 cm). Endurance of male mice (n = 4–5 per genotype) was tested at constant rotating speed (5 rpm). Motor learning of male mutants (n = 24) and controls (n = 32) was assessed with the following protocol: On day 1 (habituation) mice were placed three times on the rod (with 10 min intertrial resting intervals). Rotating speed was successively increased from 3.3–12 rpm within 2 min. On day 2 (challenge) mice were placed 5 times on a rod (with 10 min intertrial resting interval). Rotating speed was successively increased from 4.1–41 rpm within 6 min. To detect subtle motor coordination defects mutants (n = 8) and controls (n = 4) were placed on a grid (30 × 30 cm, 1,5 cm grid hole size) and allowed to freely explore. Numbers of front and hind limb slips were quantified in 2 consecutive trials (3 min each; intertrial intervals, 10 min). Balancing performance was tested by placing male mice (controls, n = 6; mutants, n = 8) three times (10 min intertrial resting intervals) on a horizontal bar (diameter 1.8 cm) for a maximum of 1 min and measuring the time before mice fell off.

### Statistical analysis

Reported values for electrophysiology and morphometry are the mean ± SEM., all other numerical values are shown as the mean ± SD. Unless stated otherwise, p values were determined by the Student’s *t* test of two-tailed uncoupled samples. In Figs 1D and 8B we used multiple *t* tests and corrected for multiple comparisons using Holm-Sidak method. Statistical differences were considered to be significant when *p* < 0.05 (*p < 0.05, **p < 0.01, ***p < 0.001). All statistical analysis was performed using the software Statistica 10.0 (StatSoft, Tulsa, USA), GraphPad (Prism) and MS Excel.

## References

[1] Armstrong DM, Schild RF (1978). An investigation of the cerebellar cortico-nuclear projections in the rat using an autoradiographic tracing method. I. Projections from the vermis. Brain Res.

[2] Yu QX, Ebner TJ, Bloedel JR (1985). Electrophysiological study of the corticonuclear projection in the cat cerebellum. Brain Res.

[3] Ito M (2006). Cerebellar circuitry as a neuronal machine. Prog Neurobiol.

[4] Hashimoto K, Kano M (2013). Synapse elimination in the developing cerebellum. Cell Mol Life Sci.

[5] Machold R, Fishell G (2005). Math1 is expressed in temporally discrete pools of cerebellar rhombic-lip neural progenitors. Neuron.

[6] Wang VY, Rose MF, Zoghbi HY (2005). Math1 expression redefines the rhombic lip derivatives and reveals novel lineages within the brainstem and cerebellum. Neuron.

[7] Chedotal AS (2010). I stay or should I go? Becoming a granule cell. Trends Neurosci.

[8] Hoshino M (2005). Ptf1a, a bHLH transcriptional gene, defines GABAergic neuronal fates in cerebellum. Neuron.

[9] Fleming JT (2013). The Purkinje neuron acts as a central regulator of spatially and functionally distinct cerebellar precursors. Dev Cell.

[10] Leto K, Rossi F (2012). Specification and differentiation of cerebellar GABAergic neurons. Cerebellum.

[11] Sotelo C (2015). Molecular layer interneurons of the cerebellum: developmental and morphological aspects. Cerebellum.

[12] Chan-Palay V, Palay SL (1972). The stellate cells of the rat’s cerebellar cortex. Z Anat Entwicklungsgesch.

[13] Rakic P (1972). Extrinsic cytological determinants of basket and stellate cell dendritic pattern in the cerebellar molecular layer. J Comp Neurol.

[14] Schilling K, Oberdick J (2009). The treasury of the commons: making use of public gene expression resources to better characterize the molecular diversity of inhibitory interneurons in the cerebellar cortex. Cerebellum.

[15] Barmack NH, Yakhnitsa V (2008). Functions of interneurons in mouse cerebellum. J Neurosci.

[16] Coddington LT, Rudolph S, Vande Lune P, Overstreet-Wadiche L, Wadiche JI (2013). Spillover-mediated feedforward inhibition functionally segregates interneuron activity. Neuron.

[17] Dizon MJ, Khodakhah K (2011). The role of interneurons in shaping Purkinje cell responses in the cerebellar cortex. J Neurosci.

[18] Jorntell H, Bengtsson F, Schonewille M, De Zeeuw CI (2010). Cerebellar molecular layer interneurons - computational properties and roles in learning. Trends Neurosci.

[19] Mittmann W, Koch U, Hausser M (2005). Feed-forward inhibition shapes the spike output of cerebellar Purkinje cells. J Physiol.

[20] Ben-Arie N (1997). Math1 is essential for genesis of cerebellar granule neurons. Nature.

[21] Yamada M (2014). Specification of spatial identities of cerebellar neuron progenitors by ptf1a and atoh1 for proper production of GABAergic and glutamatergic neurons. J Neurosci.

[22] Butts T, Hanzel M, Wingate RJ (2014). Transit amplification in the amniote cerebellum evolved via a heterochronic shift in NeuroD1 expression. Development.

[23] Miyata T, Maeda T, Lee JE (1999). NeuroD is required for differentiation of the granule cells in the cerebellum and hippocampus. Genes Dev.

[24] Olson JM (2001). NeuroD2 is necessary for development and survival of central nervous system neurons. Dev Biol.

[25] Cherry TJ (2011). NeuroD factors regulate cell fate and neurite stratification in the developing retina. J Neurosci.

[26] Brohl D (2008). A transcriptional network coordinately determines transmitter and peptidergic fate in the dorsal spinal cord. Dev Biol.

[27] Lin CH (2004). Regulation of neuroD2 expression in mouse brain. Dev Biol.

[28] Bormuth I (2013). Neuronal basic helix-loop-helix proteins Neurod2/6 regulate cortical commissure formation before midline interactions. J Neurosci.

[29] Naya FJ (1997). Diabetes, defective pancreatic morphogenesis, and abnormal enteroendocrine differentiation in BETA2/neuroD-deficient mice. Genes Dev.

[30] Goebbels S (2005). Cre/loxP-mediated inactivation of the bHLH transcription factor gene NeuroD/BETA2. Genesis.

[31] Madisen L (2010). A robust and high-throughput Cre reporting and characterization system for the whole mouse brain. Nat Neurosci.

[32] Liu M (2000). Loss of BETA2/NeuroD leads to malformation of the dentate gyrus and epilepsy. Proc Natl Acad Sci USA.

[33] Lin CH, Tapscott SJ, Olson JM (2006). Congenital hypothyroidism (cretinism) in neuroD2-deficient mice. Mol Cell Biol.

[34] Dezonne RS, Lima FR, Trentin AG, Gomes FC (2015). Thyroid hormone and astroglia: endocrine control of the neural environment. J Neuroendocrinol.

[35] Hashimoto K, Kano M (2003). Functional differentiation of multiple climbing fiber inputs during synapse elimination in the developing cerebellum. Neuron.

[36] Hashimoto K (2009). Influence of parallel fiber-Purkinje cell synapse formation on postnatal development of climbing fiber-Purkinje cell synapses in the cerebellum. Neuroscience.

[37] Hashimoto K, Ichikawa R, Kitamura K, Watanabe M, Kano M (2009). Translocation of a “winner” climbing fiber to the Purkinje cell dendrite and subsequent elimination of “losers” from the soma in developing cerebellum. Neuron.

[38] Kume H (1996). Molecular cloning of a novel basic helix-loop-helix protein from the rat brain. Biochem Biophys Res Commun.

[39] Schwab MH (1998). Neuronal basic helix-loop-helix proteins (NEX, neuroD, NDRF): spatiotemporal expression and targeted disruption of the NEX gene in transgenic mice. J Neurosci.

[40] Funfschilling U, Reichardt LF (2002). Cre-mediated recombination in rhombic lip derivatives. Genesis.

[41] Maricich SM, Herrup K (1999). Pax-2 expression defines a subset of GABAergic interneurons and their precursors in the developing murine cerebellum. J Neurobiol.

[42] Weisheit G (2006). Postnatal development of the murine cerebellar cortex: formation and early dispersal of basket, stellate and Golgi neurons. Eur J Neurosci.

[43] D’Angelo E, De Zeeuw CI (2009). Timing and plasticity in the cerebellum: focus on the granular layer. Trends Neurosci.

[44] Lujan R, Albasanz JL, Shigemoto R, Juiz JM (2005). Preferential localization of the hyperpolarization-activated cyclic nucleotide-gated cation channel subunit HCN1 in basket cell terminals of the rat cerebellum. Eur J Neurosci.

[45] Bobik M, Ellisman MH, Rudy B, Martone ME (2004). Potassium channel subunit Kv3.2 and the water channel aquaporin-4 are selectively localized to cerebellar pinceau. Brain Res.

[46] Ango F (2004). Ankyrin-based subcellular gradient of neurofascin, an immunoglobulin family protein, directs GABAergic innervation at purkinje axon initial segment. Cell.

[47] Hausser M, Clark BA (1997). Tonic synaptic inhibition modulates neuronal output pattern and spatiotemporal synaptic integration. Neuron.

[48] Llano I, Gerschenfeld HM (1993). Inhibitory synaptic currents in stellate cells of rat cerebellar slices. J Physiol.

[49] Doetschman T (2009). Influence of genetic background on genetically engineered mouse phenotypes. Methods Mol Biol.

[50] Buss RR, Sun W, Oppenheim RW (2006). Adaptive roles of programmed cell death during nervous system development. Annu Rev Neurosci.

[51] Dressler GR (1993). Deregulation of Pax-2 expression in transgenic mice generates severe kidney abnormalities. Nature.

[52] Cioni JM (2013). SEMA3A signaling controls layer-specific interneuron branching in the cerebellum. Curr Biol.

[53] Llano I (2000). Presynaptic calcium stores underlie large-amplitude miniature IPSCs and spontaneous calcium transients. Nat Neurosci.

[54] Nakayama H (2012). GABAergic inhibition regulates developmental synapse elimination in the cerebellum. Neuron.

[55] Hausser M, Clark BA (1997). Tonic synaptic inhibition modulates neuronal output pattern and spatiotemporal synaptic integration. Neuron.

[56] Bäurle J, Grüsser-Cornehls U (1994). Axonal torpedoes in cerebellar Purkinje cells of two normal mouse strains during aging. Acta Neuropathol..

[57] Ljungberg L (2016). Transient developmental Purkinje cell axon torpedoes in healthy and ataxic mouse cerebellum. Front Cell Neurosci..

[58] De Zeeuw CI (2011). Spatiotemporal firing patterns in the cerebellum. Nat Rev Neurosci.

[59] Wulff P (2009). Synaptic inhibition of Purkinje cells mediates consolidation of vestibulo-cerebellar motor learning. Nat Neurosci.

[60] Palay, S. L. & Chan-Palay, V. *Cerebellar cortex: cytology and organization*. (Springer 1974).

